# Sex Differences in Neuropsychological Functioning are Domain-Specific in Adolescent and Young Adult Regular Cannabis Users

**DOI:** 10.1017/S1355617720001435

**Published:** 2021-07

**Authors:** George Savulich, Natali Rychik, Erin Lamberth, Maya Hareli, A. Eden Evins, Barbara J. Sahakian, Randi M. Schuster

**Affiliations:** 1 Department of Psychiatry and Behavioral and Clinical Neuroscience Institute, University of Cambridge, School of Clinical Medicine, Cambridge, UK; 2 Center for Addiction Medicine, Department of Psychiatry, Massachusetts General Hospital (MGH), Boston, MA, USA; 3 Department of Psychology, Loyola University Chicago, Chicago, IL, USA; 4 Harvard Medical School, Boston, MA, USA

**Keywords:** Cannabis, marijuana, sex, neurodevelopment, neuropsychology, adolescence

## Abstract

**Objective:**

Adolescence into young adulthood represents a sensitive period in which brain development significantly diverges by sex. Regular cannabis use by young people is associated with neuropsychological vulnerabilities, but the potential impact of sex on these relationships is unclear.

**Method:**

In a cross-sectional study, we examined sex differences in multi-domain neuropsychological functioning using the Cambridge Neuropsychological Test Automated Battery (CANTAB) and tested whether sex moderated the relationship between cognitive performance and age of initiation, frequency of cannabis use, amount of cannabis use, and withdrawal symptoms in at least weekly adolescent and young adult cannabis users (*n* = 171; aged 13–25 years; 46.2% female).

**Results:**

Male cannabis users had poorer visual recognition memory and female cannabis users showed worse attention and executive functions, with medium to large effect sizes. These sex effects persisted, when controlling for age, IQ, amount of alcohol and nicotine use, mood and anxiety symptoms, emotional stability and impulsive behavior. Earlier age of initiated use and more use were associated with worse attentional functions in females, but not males. More use was more strongly associated with worse episodic memory in males than in females. More use was associated with poorer learning in males only.

**Conclusions:**

Domain-specific patterns of neuropsychological performance were found by sex, such that males showed poorer visual memory and females showed worse performance on measures of attention (sustained visual, multitasking) and executive functioning (spatial planning/working memory subdomains). Larger studies including healthy controls are needed to determine if the observed sex differences are more exaggerated relative to non-users.

## INTRODUCTION

Cannabis is one of the most widely used substances in the world, and is one of the most commonly used substances among adolescents and young adults in high-income countries (Hall & Degenhardt, 2009). In the United States, 12.5% of adolescents aged 12 to 17 years and 34.8% of young adults aged 18 to 25 years used cannabis in the past year (Substance Abuse and Mental Health Services Administration, 2019), with around 5% of high school students reporting daily use (Johnston et al., 2020). The continued legalization and commercialization of cannabis for medicinal and recreational purposes has resulted in decreased perceptions of harm and increased acceptability and availability even among youth (Cohen, 2010). In heavy adult users, poor cognitive performance has been reported in multiple domains including attention, executive functions, psychomotor speed, episodic memory, and verbal fluency (e.g. Lisdahl & Tapert, 2012), with accumulating evidence indicating sex differences (Crane, Schuster, Fusar-Poli, & Gonzalez, 2013; Lisdahl & Price, 2012; King et al., 2011; Clark, Roiser, Robbins, & Sahakian, 2009; Pope, Jacobs, Mialet, Yurgelun-Todd, & Gruber, 1997). However, surprisingly few studies have examined how chronic cannabis use may differentially impact neuropsychological functioning in male and female cannabis users in adolescence and young adulthood.

Adolescence into young adulthood is a vulnerable period for brain development, characterized by profound and dynamic changes in cortical areas, the limbic system, and white matter tracts associated with higher order cognitive and affective processes (Bava & Tapert, 2010). Gray matter volume peaks around the ages of 12 and 14 years and then declines throughout adolescence, first in primary sensorimotor cortices and lastly in the dorsolateral prefrontal cortex (Giedd et al., 1999; Gogtay et al., 2004; Gogtay & Thompson, 2010; Sowell, Thompson, Holmes, Jernigan, & Toga, 1999). During adolescence, increased myelination and synaptic pruning occurs (Sowell, Thompson, Tessner, & Toga, 2001; Huttenlocher & Dabholkar, 1997), with white matter volume peaking in mid-life (Bartzokis et al., 2010). The endocannabinoid system also changes during this period with significant pruning of receptor density in subcortical and frontal regions (Heng, Beverley, Steiner, & Tseng, 2011). Brain regions centrally involved in higher-order cognitive functions and with a high expression of cannabinoid receptors (CB1) may thus be particularly susceptible to insults from exogenous cannabinoids (Herkenham et al., 1990; Mackie, 2005; Van Laere et al., 2008). There is evidence that sex moderates the impact of adolescent cannabis exposure on brain structure, with female users aged 16 to 19 years reported to show larger amygdala volume compared with male and female non-users (Mcqueeny et al., 2011). In female users aged 16 to 18 years, larger volume of the prefrontal cortex (PFC) has been reported to have an association with poorer executive functioning specifically (Medina et al., 2009).

Previous research has focused on the effects of heavy cannabis use on neuropsychological functioning in adolescents and young adults, with studies showing small overall effect sizes (Scott et al., 2018). Nonetheless, cognitive difficulties have been shown to improve with abstinence (Schuster et al., 2018; Wallace, Wade, & Lisdahl, 2020), supporting their association with continued use. In young adults, sex has been reported to moderate the association between cognitive dysfunction and the frequency (Lisdahl & Price, 2012) and amount (Crane, Schuster, & Gonzalez, 2013) of cannabis use, as well as the age of initiated use (Crane, Schuster, Mermelstein, & Gonzalez, 2015). Several studies have supported associations between earlier age of cannabis use onset and worse neuropsychological outcomes (e.g. Battisti et al., 2010; Ehrenreich et al., 1999; Fontes et al., 2011; Gruber, Sagar, Dahlgren, Racine, & Lukas, 2012; Schuster, Hoeppner, Evins, & Gilman, 2016). Sex may also moderate the severity of withdrawal from cannabis. For example, females report greater incidence and severity of withdrawal symptoms and experience more difficulty achieving abstinence than males (Cooper & Craft, 2018; Herrmann, Weerts, & Vandrey, 2015). However, the impact of sex on the relationship between withdrawal severity and neuropsychological functioning remains to be clarified.

We investigated the relationship between sex, cannabis exposure levels, and cognitive performance using a standardized comprehensive test battery, controlling for key demographic and drug-related variables (i.e. age, IQ, amount of alcohol and nicotine use in the past 90 days, mood and anxiety symptoms, emotional stability, and impulsive behavior) in a community sample of high school and college students aged 13 to 25 years. Based on previous evidence showing sex-specific disruption of CB1 receptor signaling by repeated cannabis exposure (Burston, Wiley, Craig, Selley, & Sim-Selley, 2010), prefrontal structural differences between sexes (Medina et al., 2009), and sex differences in neuropsychological functioning among adult cannabis users (e.g. Lisdahl & Price, 2012; King et al., 2011; Pope et al., 1997), we hypothesized that female adolescents who used cannabis would demonstrate worse cognitive performance than males, particularly for functions subserved by frontal-limbic circuitry. In addition, we tested whether sex moderated the relationship between neuropsychological functioning and age of initiation of regular cannabis use, frequency of cannabis use in the past 90 days, amount of cannabis use in the past 90 days, and on cannabis withdrawal symptoms in this sample.

## METHOD

### Participants

This study evaluated the baseline wave of data collection from two longitudinal trials of cannabis use and cognition, one involving participants aged 18–25 years (Cohort 1 *n* = 88; 50 male, 38 female) and one involving those aged 13–19 years (Cohort 2 *n* = 83; 42 male, 41 female), both of whom reported using cannabis at least weekly (i.e. at minimum once per week, most weeks, and at least once within 7 days of baseline). The primary aim of the parent studies is to evaluate the impact of cannabis abstinence on neuropsychological functioning in a community sample of adolescents. Participants were recruited from community advertisements, peer referrals, and school surveys administered in the local Boston community, and screened for eligibility by telephone. Inclusion criteria included English language fluency and at least weekly cannabis use. Participants were excluded for severe developmental delays likely to impact neuropsychological functioning, including but not limited to autism spectrum disorder, intellectual disability, and Down’s syndrome. All participants were asked to refrain from all substance use on the day of their study visit, with the exception of nicotine, caffeine, and prescription drug use. Participants over the age of 18 provided written informed consent. For participants under the age of 18, written informed assent was obtained as was written informed consent from a parent or legal guardian. Protocols were approved by the Partners Human Research Committee Institutional Review Board.

## MEASURES

### Substance Use

Frequency of cannabis and alcohol use in the past 90 days (cumulative number of days used), amount of cannabis, alcohol and nicotine use in the past 90 days (total number of grams of cannabis,^1^ number of alcoholic drinks and number of cigarettes), and age of initiation were assessed using a modified Timeline Follow-Back method (Robinson et al., 2014). Intensity and negative impact of cannabis withdrawal were assessed with the Cannabis Withdrawal Scale (CWS; Allsop, Norberg, Copeland, Fu, & Budney 2011; Cronbach’s *α* = 0.91). Cannabis and alcohol dependence were assessed using the Cannabis Use Disorder Identification Test-Revised (CUDIT-R; Adamson & Sellman, 2003; Cronbach’s *α* = 0.91) and Alcohol Use Disorders Identification Test (AUDIT; Saunders et al., 1993; Cronbach’s *α* = 0.81). Participants provided a urine sample on the day of their study visit to quantitatively ascertain levels of creatinine-adjusted 11-nor-9-carboxy-Δ9-tetrahydrocannabinol metabolite levels via liquid chromatography–tandem mass spectrometry (LC/MS/MS) and were qualitatively screened with an immunoassay rapid dip drug test (RDDT; Medimpex United Inc.) for the presence of cannabis, cocaine, opiates, amphetamine, and methamphetamine.

### Psychopathology

Current Axis 1 psychiatric disorders (including substance use disorders) were assessed via semi-structured interview (Cohort 1: Structured Clinical Interview for DSM-5, SCID-5; First et al., 2015; and Kiddie Schedule for Affective Disorders and Schizophrenia, K-SADS; Endicott & Spitzer, 1978; Cohort 2: Mini-International Neuropsychiatric Inventory for Children and Adolescents, MINI Kid; Sheehan et al., 1998). Current and childhood symptoms of attention deficit/hyperactivity disorder (ADHD) were assessed with a DSM-5 symptom checklist (American Psychiatric Association, 2013). Past week symptoms of anxiety and depression were assessed with the Mood and Anxiety Symptom Questionnaire (MASQ; Watson et al., 1995; Cronbach’s *α* = 0.85–0.93).

### Trait Measures

Personality was assessed with the 10-Item Personality Measure (TIPI; Gosling, Rentfrow, & Swann, 2003; Cronbach’s *α* = 0.40–0.73) and impulsive behavior was assessed with the Urgency, Premeditation (lack of), Perseverance (lack of), Sensation Seeking, Positive Urgency, Impulsive Behavior Scale (UPPS-P; Cyders & Smith, 2007; Cronbach’s *α* = 0.80–0.94).

### Neuropsychological Functioning

Full-scale IQ was estimated using the Wechsler Test of Adult Reading (WTAR; Wechsler, 1999) in Cohort 1 and the two-subset Wechsler Abbreviated Scale of Intelligence (WASI; Wechsler, 2001) in Cohort 2. Neuropsychological functioning was assessed using the CANTAB (www.cambridgecognition.com), a reliable and well-validated battery that has been previously used in populations with drug use, including cannabis (e.g. Meier et al., 2012; Grant, Chamberlain, Schreiber, & Odlaug, 2012). CANTAB Research Suite (Windows-based) was administered in Cohort 1 and CANTAB Connect (iPad-based) was administered in Cohort 2; both platforms are touchscreen, with tasks showing good test–retest reliability (Pearson correlation coefficients ≥ 0.72) (Cambridge Cognition, internal data).

### Memory

The Delayed Matching to Sample (DMS) task assesses visual matching and short-term visual recognition memory (Robbins et al., 1997) (Cohort 1 only). Participants are shown an abstract, non-verbal pattern (the sample) followed by four similar patterns simultaneously and after 4000 and 12000 ms delays. Participants are then asked to select the pattern that matches the sample. Outcome measures include the percentage of correct responses and mean response latency (ms) at all three delay intervals.

The Paired Associates Learning (PAL) task assesses episodic memory and new learning (Sahakian et al., 1988) (Cohort 2 only). Boxes are displayed on the screen and opened in a randomised order, some of which contain a pattern. The patterns are then displayed in the middle of the screen, one at a time, and the participant must touch the box where they think the pattern was originally located. If a participant makes an error, the patterns are presented again. Outcome measures include the total number of errors made (a measure of new learning) and first trial memory score (i.e. the number of patterns correctly located after the first trial summed across the number of stages completed).

The Verbal Recognition Memory (VRM) task assesses ability to encode and retrieve verbal information (Fletcher & Henson, 2001) (Cohort 2 only). Eighteen words are presented, and participants are subsequently asked to identify them among distractor words immediately and after a 20-min delay. Outcome measures include the number of correct responses at immediate and delayed recall.

### Attention and Executive Functions

The Rapid Visual Information Processing (RVP) task assesses sustained visual attention (Pironti et al., 2014) (Cohorts 1 and 2). Single digits appear in a white box in the center of the screen at a rate of 100 digits per minute. Participants must detect a series of three target sequences (e.g. 3, 5, 7) by pressing a button as quickly as possible. Outcome measures include mean response latency (ms) on correctly identified targets and A’ (sensitivity to detecting the target sequence regardless of response tendency).

The Multitasking Test (MTT) assesses ability to manage conflicting information. Arrows appear on either side of the screen (left/right) and point in either direction (to the left or right) (Cohorts 1 and 2). A cue at the top of the screen indicates whether the participant should select the right or left button according to either the side on which the arrow appeared or the direction in which the arrow was pointing. In some sections of the task the same rule is applied consistently (either side or direction, single task), whereas in others the rules are presented in a randomized order (multitasking). Outcome measures include switching cost (the difference between response latencies when the rule was switching *vs.* when the rule remained constant) and congruency cost (the difference between response latency of congruent *vs.* incongruent trials) for correct responses only.

The One Touch Stockings of Cambridge (OTS) task assesses spatial planning and working memory (Owen, Downes, Sahakian, Polkey, & Robbins, 1990) (Cohorts 1 and 2). Participants are shown two displays containing three colored balls. Participants must work out the minimum number of moves required to copy the upper display (by moving the balls in the lower display to a new position) and select their response from a row of numbered boxes along the bottom of the screen. Outcome measures include mean choices to correct and the number of problems solved on the first choice.

The Spatial Working Memory (SWM) assesses ability to retain spatial information and manipulate remembered items in working memory (Owen et al., 1990) (Cohort 2). Colored boxes first appear on the screen, and participants are instructed to find a yellow token in each box using a process of elimination. The position of the boxes changes and the number of boxes increases for each trial. Outcome measures include total errors (selecting boxes that have already been found to be empty and/or revisiting boxes which have already been found to contain a token) and strategy (a predetermined sequence by beginning with a specific box and then, once a token has been found, returning to that box to start a new search sequence).

### Statistical Analyses

Analyses were conducted using SPSS 25.0. Demographic, mental health, substance use, and personality trait measures were analyzed by sex using independent samples *t*-tests or chi-square tests as appropriate. Frequency and amount of cannabis and alcohol use were analysed by sex using Mann–Whitney *U* tests given their highly skewed distributions. Males and females were compared on CANTAB outcome measures using analysis of covariance for each task separately, controlling for age, estimate of intelligence (IQ), amount of alcohol (number of drinks) and nicotine (number of cigarettes) use in the past 90 days, mood and anxiety symptoms (MASQ subscales), emotional stability (TIPI subscale), and impulsive behavior (UPPS-P perseverance and sensation seeking subscales). The Benjamini–Hochberg procedure was applied at *q* < 0.10 to control for false discovery; all significant *p*-values remained (two-sided).

We then explored potential interactions (separately) with age of initiated use, frequency of cannabis use in the past 90 days (number of days), amount of cannabis use in the past 90 days (grams consumed), and withdrawal symptoms to test if similar patterns of cognitive performance were also related to measures of use severity. As in previous studies (Crane, Schuster, Fusar-Poli, et al., 2013; Crane, et al., 2015), we conducted a moderated hierarchical multiple regression analysis with neuropsychological performance as the dependent variable and age of cannabis initiation, frequency of cannabis use, amount of cannabis use, and cannabis withdrawal subscales (intensity, negative impact of withdrawal) as predictors (separately) in Block (model) 1; vectors for sex (male, female) in Block 2; and their interaction in Block 3. We additionally controlled for the same covariates delineated above (entered in the first block) to better isolate any observed effects to the influence of sex. Interdependencies between covariates were examined using multicollinearity diagnostics. Variance inflation factors ranged between 1.1 and 5.4 for each variable and were thus regarded as acceptable.

## RESULTS

### Demographic, Mental Health, Trait, and Substance Use Measures

The groups did not differ significantly in age, IQ, or scholastic achievement. Females reported more than twice the rate of anxiety disorders and significantly greater symptoms on MASQ subscales than males. Males reported greater emotional stability, more perseverance, and higher sensation-seeking than females (Table 1).

On substance use measures, males had significantly higher AUDIT scores and a trend for higher CUDIT scores, and reported higher amount of use for alcohol, cannabis and nicotine though not significantly more days using either substance (alcohol or cannabis) or days since last cannabis or nicotine use. Females reported more negative impact of cannabis withdrawal and a trend for greater intensity of withdrawal (Table 2).

### Neuropsychological Performance

Univariate analysis of covariance revealed *domain-specific* patterns of cognitive performance by sex. Males had slower response latencies (DMS) than females after the 4000 and 12000 delay intervals (i.e. poorer visual recognition memory). Females showed greater switching cost (MTT), poorer target sensitivity (RVP A’) (i.e. worse sustained visual attention), and had a less efficient search strategy (SWM) than males (Table 3).

### Relationship between Sex, Age of Initiated Use, and Neuropsychological Performance

**Fig. 1.**
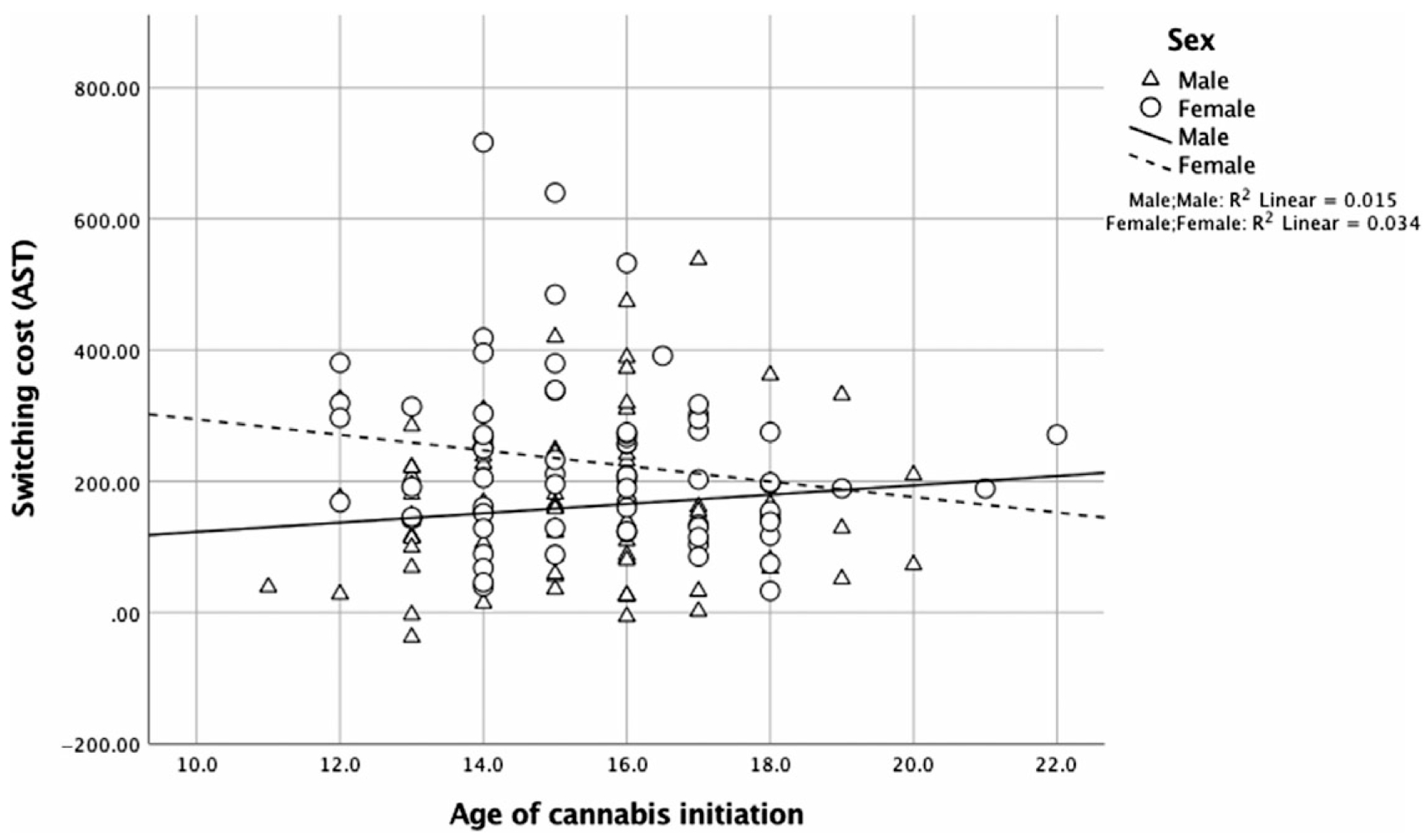
For switching cost (MTT), the interaction between sex and age of cannabis initiation did not reach significance, [*R^2^* = .21, *β* = −1.13, *p* = .07, 95% CI (−36.60, 1.12)]. Earlier age of first cannabis use was associated with greater switching cost for females, [*β* = −0.30, *p* = .04, 95% CI (−36.53, −1.23)], but not for males, [*β* = 0.02, *p* = .88, 95% CI (−13.41, 15.62)].

For switching cost on the multitasking test of attention (MTT), there were main effects of age, [*β* = 0.22, *p* = .02, 95% CI (1.54, 21.52)] and sex, [*β* = 1.43, *p* = .02, 95% CI (61.77, 649.39)] (see Table 4 for main effects and interactions on all neuropsychological outcomes). The interaction between sex and age of cannabis initiation did not reach significance, [*R^2^* = .21, *β* = −1.13, *p* = .07, 95% CI (−36.60, 1.12)] (Figure 1). In analyses of simple slopes, earlier age of first cannabis use was associated with greater switching cost for females, [*β* = −0.30, *p* = .04, 95% CI (−36.53, −1.23)], but not for males, [*β* = 0.02, *p* = .88, 95% CI (−13.41, 15.62)]. There was no significant interaction of age of cannabis initiation with sex for other neuropsychological outcomes (*p*-values > .11).

### Relationship between Sex, Frequency and Amount of Cannabis Use, and Neuropsychological Performance

**Fig. 2.**
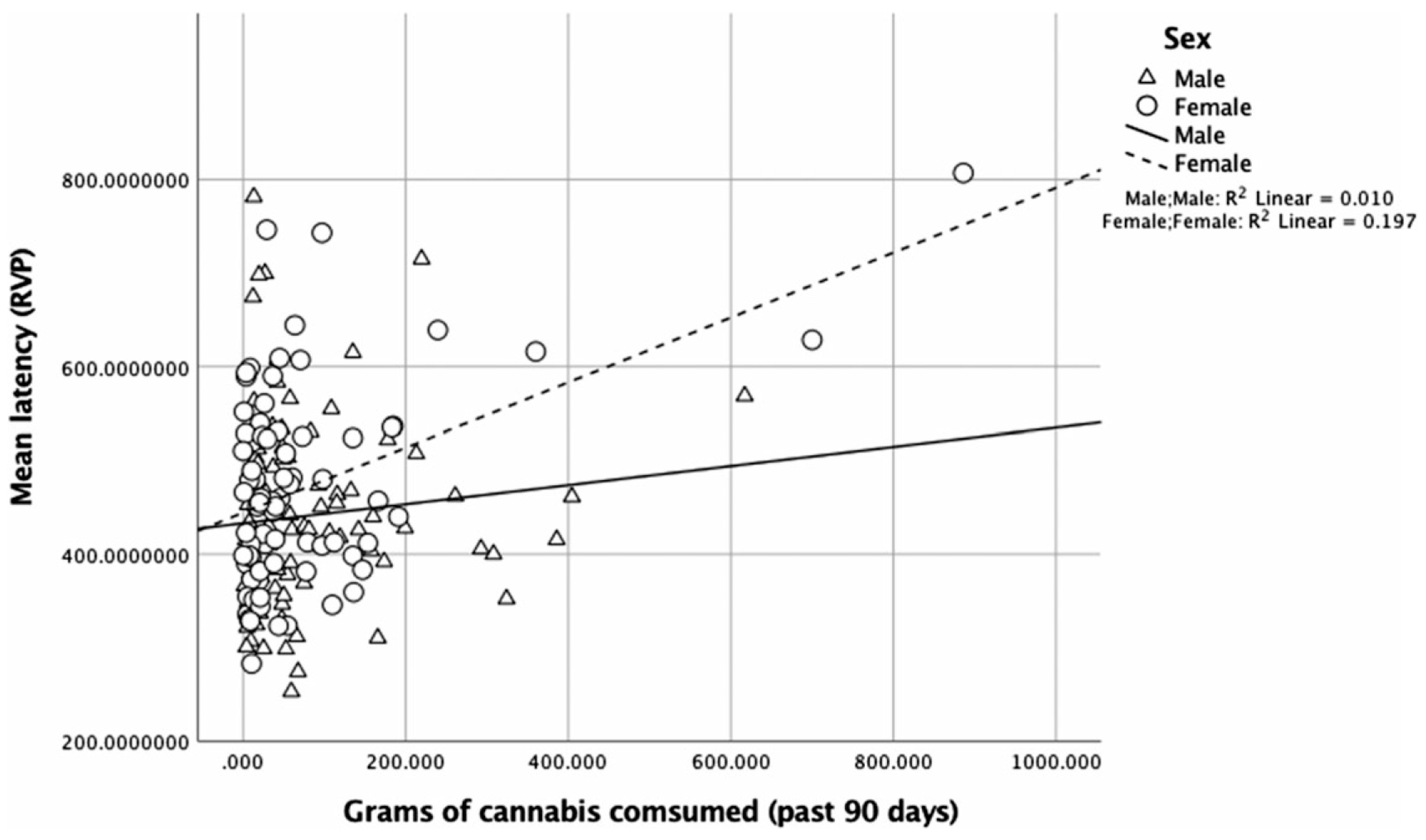
For mean response latency (RVP), the interaction between sex and amount of cannabis use in the past 90 days was significant, [*R^2^* = .24, *β* = 0.28, *p* = .02, 95% CI (0.05, 0.55)]. More use was associated with slower response latency for females, [*β* = 0.35, *p* = .004, 95% CI (0.92, 0.45)], but not for males, [*β* = 0.04, *p* = .72, 95% CI (−0.19, 0.27)].

There were no significant interactions of frequency of cannabis use in the past 90 days with sex for neuropsychological outcomes (*p*-values > .18). For mean response latency on the task of sustained visual attention (RVP), there was a main effect of age, [*β* = −0.27, *p* < .001, 95% CI (597.06, 1143.49)], and the interaction between sex and amount of cannabis use in the past 90 days was significant, [*R^2^* = .24, *β* = 0.28, *p* = .02, 95% CI (0.05, 0.55)] (Figure 2). In analyses of simple slopes, more cannabis use was associated with slower response latency for females, [*β* = 0.35, *p* = .004, 95% CI (0.92, 0.45)], but not for males, [*β* = 0.04, *p* = .72, 95% CI (−0.19, 0.27)].

**Fig. 3.**
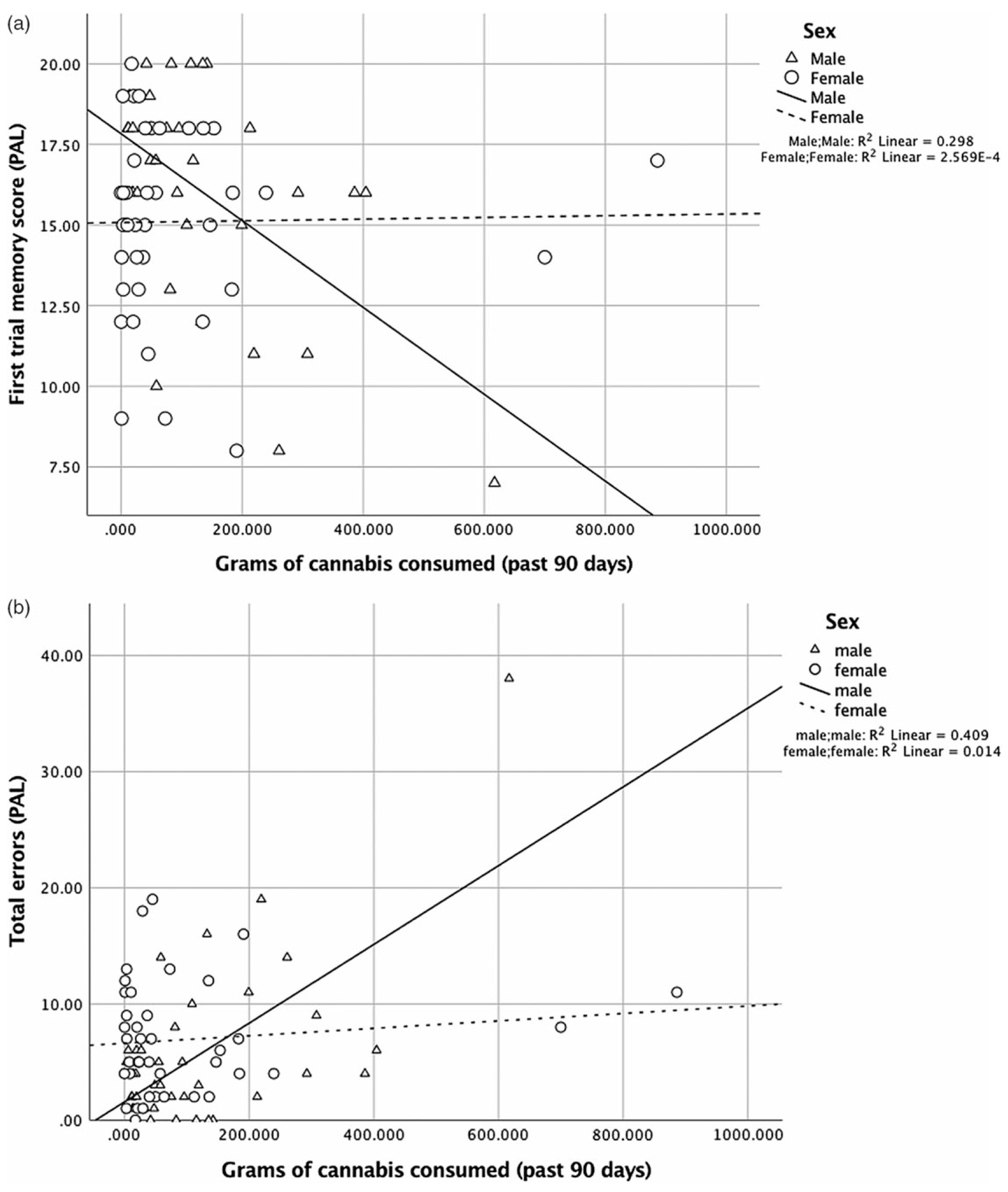
(**A**) For first trial memory score (PAL), the interaction between sex and amount of cannabis use in the past 90 days was significant, [*R^2^* = .29, *β* = 0.68, *p* = .001, 95% CI (0.01, 0.03)]. More use was more strongly associated with worse episodic memory in males [*β* = −0.61, *p* < .001, 95% CI (−0.02, −0.01)] than in females [*β* = 0.42, *p* = .04, 95% CI (0.00, 0.01)]. (**B**) For total errors (PAL), the interaction between sex and amount of cannabis use in the past 90 days was significant, [*R^2^* = .40, *β* = −0.78, *p* < .001, 95% CI (−0.05, −0.02)]. More use was associated with poorer learning (total errors) for males [*β* = 0.72, *p* < .001, 95% CI (0.03, 0.05)], but not for females [*β* = −0.24, − = .22, 95% CI (−0.02, 0.004)]. The pattern of results was the same when removing cases more than 3 * IQR (interquartile range) (memory score: males: *β* = 0.42, *p* = .02, females: *β* = 0.42, *p* = .04; errors: males: *β* = 0.39, *p* = .04, females: *β* = −0.24, *p* = .22)

For the PAL first trial memory score, there were main effects of sex, [*β* = −0.48, *p* = .002, 95% CI (−4.84, −1.10)] and amount of use, [*β* = −0.64, *p* = .001, 95% CI (−0.02, −0.01)], and their interaction was significant, [*R^2^* = .29, *β* = 0.68, *p* = .001, 95% CI (0.01, 0.03)] (Figure 3A). Similarly for PAL total errors made, there were main effects of sex, [*β* = 0.46, *p* = .002, 95% CI (2.18, 8.84)] and amount of use, [*β* = 0.88, *p* < .001, 95% CI (0.02, 0.05)], and their interaction was also significant, [*R^2^* = .40, *β* = −0.78, *p* < .001, 95% CI (−0.05, −0.02)] (Figure 3B). Analyses of the simple slopes showed that more cannabis use was more strongly associated with worse episodic memory in males [memory score: *β* = −0.61, *p* < .001, 95% CI (−0.02, −0.01)] than in females [memory score: *β* = 0.42, *p* = .04, 95% CI (0.00, 0.01)]. More cannabis use was associated with poorer learning (total errors) in males only [males: *β* = 0.72, *p* < .001, 95% CI (0.03, 0.05)]; [females: *β* = −0.24, *p* = .22, 95% CI (−0.02, 0.004)].

### Relationship between Sex, Withdrawal, and Neuropsychological Performance

For OTS mean choices to correct, there were main effects of IQ, [*β* = −0.33, *p* < .001, 95% CI (−0.01, −0.01)] and anxious arousal, [*β* = 0.32, *p* = .01, 95% CI (0.004, 0.02)], and the interaction between sex and negative impact of cannabis withdrawal was significant. [*R^2^* = .24, *β* = 0.40, *p* = .02, 95% CI (0.001, 0.009)]. For OTS number of problems solved, there were main effects of IQ, [*β* = 0.19, *p* = .03, 95% CI (0.004, 0.07)], cigarette use in the past 90 days, [*β* = −0.20, *p* = .01, 95% CI (−0.01, −0.001)], anxious arousal, [*β* = −0.32, *p* = .01, 95% CI (−0.17, −0.02)], and perseverance, [*β* = 0.22, *p* = .02, 95% CI (0.02, 0.16)], and the interaction between sex and negative impact of cannabis withdrawal was significant, [*R^2^* = .20, *β* = −0.40, *p* = .02, 95% CI (−0.06, −0.01)]. However, analyses of the simple slopes did not support sex as a notable factor (*p*-values > .34). There were no significant sex by withdrawal intensity interactions for neuropsychological outcomes (*p*-values > .11).

## DISCUSSION

In a community sample of adolescent and young adult regular cannabis users, we found domain-specific patterns of cognitive performance by sex, such that males showed poorer visual recognition memory and females showed worse performance on measures of attention (sustained visual, multitasking) and executive functions (i.e. spatial planning/working memory subdomains). Exploratory analyses further suggested that earlier age of initiated use was associated with poorer ability to multitask for females, but not for males, and that more cannabis use was associated with slower response latency for females, but not for males. Exploratory analyses also showed that sex interacted with cannabis use in the past 90 days to moderate memory performance. Specifically, more cannabis use was more strongly associated with worse episodic memory (i.e. the total number of patterns correctly located after the first trial) in males than in females. More use was associated with worse learning (i.e. the total number of errors made across all trials attempted) in males, but not females.

### Domain-Specific Sex Differences in Neuropsychological Performance

Between-group comparisons in our sample suggested that sex differences in neuropsychological functioning are domain specific. Nonetheless, we can only draw conclusions about our cannabis-using sample by sex and cannot compare performance relative to non-users. Previous studies examining sex differences in neuropsychological functioning have found that in general, healthy, non-cannabis using males show better decision-making, spatial processing, and sensorimotor/motor speed (e.g. Reavis & Overman, 2001; Gur et al., 2012), and healthy, non-cannabis using females show better attention, reasoning, verbal and episodic memory (Gur et al., 2010; Kramer, Delis, & Daniel, 1988; Asperholm, Högman, Rafi, & Herlitz, 2019). A *reverse* pattern of cognitive performance was partly found in our study, such that female cannabis users showed poorer attentional functions. Similar sex-specific patterns of reverse association have been previously reported in non-acute studies, with females showing poorer episodic memory and males showing poorer decision-making, suggesting that cannabis use may blunt those domains in which healthy males and females typically perform better (Crane, Schuster, Mermelstein, et al., 2015).

### Attentional Dysfunction in Female Cannabis Users

Our data provide some support of prior studies showing a negative relationship between earlier age of initiated use and poorer neuropsychological functioning, including measures of attention (e.g. Battisti et al., 2010; Ehrenreich et al., 1999; Fontes et al., 2011; Gruber, Sagar, Dahlgren, Racine, & Lukas, 2012). Others have reported that attentional problems persist only in female users in early adolescence (i.e. <17 years) even after controlling for time-varying confounds (Pardini et al., 2015). In our study, switching cost (MTT) was significantly worse for females than for males, although the interaction between sex and age of initiation did not reach significance. However, more cannabis use was highly associated with slower response latency (RVP) for females only, indicating that greater cannabis exposure specifically interacts with a key indicator of sustained attentional function. Although attentional problems in early development are more characteristic of healthy, non-cannabis using males, we speculate that self-treatment of potentially undiagnosed attentional problems may drive earlier and greater use in cannabis-using females (current inattention trended toward, but did not reach, significance in the current sample).

Our findings suggested sex-specific disruption to frontal-limbic circuitry most affected by cannabis use (Crane, Schuster, & Gonzalez, 2013; Martin-Santos et al., 2010). Worse attention in females is consistent with both human and animal studies. For example, preclinical evidence has shown that females selectively metabolize THC to its most active compound (Narimatsu, Watanabe, Yamamoto, & Yoshimura, 1991) and show widespread desensitization of CB1 receptors in key areas including the PFC (Burston et al, 2010). Neuroimaging evidence has shown PFC-related executive dysfunction specifically in females (Medina et al., 2009). Neuromaturation occurs in a “back to front” direction, such that the PFC is among the most susceptible regions to repeated exogeneous cannabinoid exposure during development (Gogtay & Thompson, 2010). As PFC volume peaks 1 to 2 years earlier in females (Giedd et al., 1999), it seems tenable that males might perform cognitively worse than females, given their protracted development of higher-order prefrontal areas. However, others have reported *disruption* to gray matter pruning in females (Medina et al., 2009). Interruption to pruning at later stages of development, combined with higher availability of CB1 receptors in similar regions that increase with age (Van Laere et al., 2008), might underlie the sex differences observed here.

### Association between Amount of Cannabis Use and Memory Function

Sex significantly interacted with amount of cannabis use to moderate memory performance. Specifically, we found that more use was more strongly associated with worse episodic memory in males than in females. More use was also associated with poorer learning in males, but not females. For both PAL measures (memory score, total errors), the interaction between sex and amount of use was highly significant and without influence from confounding variables, indicating sex-specific effects. These findings replicate previous studies reporting poorer memory processes with more use (e.g. Bolla, Brown, Eldreth, Tate, & Cadet, 2002; Solowij et al., 2002). With respect to sex differences, a previous study reported that greater lifetime cannabis use was more strongly associated with poorer episodic memory in females than in males (Crane, Schuster, Fusar-Poli, et al., 2013). Another study reported an association between earlier age of initiated use and poorer episodic memory, particularly immediate recall, for females only (Crane et al., 2015). It is important to note that both of these studies assessed verbal episodic memory (using the Hopkins Verbal Learning Test-Revised; Benedict, Schretlen, Groninger, & Brandt, 1998) rather than visual. Sex differences in verbal recognition memory (VRM) were not found in our sample. This could be due to differences between the tasks used, such as word lengths, semantic categorization, the number of learning trials, and/or the method of delivery (i.e. words presented verbally *vs.* read on a computer screen). Nonetheless, our findings suggest that the interaction between cannabis exposure and visual episodic memory differentially affects males and females, thus highlighting the need for sex-specific behavioral interventions, such as those that explicitly target new learning in males.

### Strengths, Limitations and Future Directions

As findings from adult studies are unlikely to generalize to younger populations, our study evaluated sex differences in neuropsychological functioning in a large, generalizable sample of adolescent and young adult regular cannabis users. We also carefully controlled for a wider array of covariates than in prior studies to better isolate the role of sex. Perhaps most importantly, including a similar proportion of male and female participants allowed for critical examination of sex-moderated neuropsychological differences in line with NIDA-supported drug research initiatives (Wetherington, 2007).

The main limitation of our study is the lack of a non-using control group. As such, it is not possible to determine if cognitive performance in our sample was weaker relative to non-users. It is also not possible from the cross-sectional design to determine whether sex differences in neuropsychological functioning predated or emerged as a consequence of regular cannabis use. Similarly, we cannot draw conclusions about the long-term effects of sex on neuropsychological functioning. We also did not exclude history of neurological illnesses (unless it precluded safe participation, e.g. epilepsy) or traumatic brain injury, which could affect cognitive performance. Finally, we acknowledge that despite controlling for mood and anxiety symptoms, this may not fully account for confounding effects of current anxiety disorders among female users on attentional performance.

Avenues of additional research include defining the hormonal, metabolic, and neuronal mediators of the observed sex differences in neuropsychological functioning. Future research should also explore if sex differences in neuropsychological functioning are more exaggerated in non-acute compared with acute samples, and whether they persist, improve, or reverse with abstinence. Following the long-term implications of these differences is of clinical significance to determine, for example, if they predict cannabis-use trajectories over time, transition to addiction, and/or treatment response. Longitudinal exploration of cognitive functioning and its relationship with age of initiation, severity of use, and withdrawal symptoms would also have strong practical utility.

## CONCLUSIONS

As cannabis legalization becomes more normative, it is important to understand its influence on the developing brain, including potential harmful effects. Our study showed that sex differences in neuropsychological functioning are domain-specific, which may reflect sex-related disruptions in frontal-limbic circuitry that emerge prior to or as a result of regular, early-onset cannabis use. Larger studies including healthy controls are needed to confirm the specificity of these effects to cannabis use, their causality, and critically, whether the neuropsychological outcomes observed in our sample are weaker compared with non-users. Sex-specific behavioral interventions may be beneficial for young cannabis users, including those that take into account dimorphic cognitive outcomes as well as diverse reasons for initiated use. As neuromaturation continues into the third decade of life, longitudinal and multimodal neuroimaging studies investigating the differential impact of cannabis on neuropsychological sequelae from pre-adolescence to young adulthood are needed in males and females separately.

## Figures and Tables

**Table 1.** Participant characteristics, mental health, substance use and trait measures by sex (means and standard deviations)

	Males *n* = 92	Females *n* = 79	Statistic, *p*-value, Effect Size
Demographics/Academic Achievement			
Age (Years)	19.64 (2.29)	19.83 (2.53)	*t* (169) = −0.51, *p* = .61, *d* = 0.08
Body Mass Index (BMI)	24.18 (3.92)	24.39 (5.28)	*t* (169) = −0.30, *p* = .77, *d* = 0.05
Verbal IQ (WTAR/WASI^a^)	107.93 (9.75)	107.46 (12.98)	*t* (169) = 0.28, *p* = .78, *d* = 0.04
Handedness (Right/ Left/ Ambidextrous)	71/ 11/ 4	68/ 6/ 1	*x^2^* = 2.60, *p* = .27, *V* = 0.13
GPA (on a 4.00 scale)	3.06 (0.49)	3.19 (0.62)	*t* (146) = −1.38, *p* = .17, *d* = 0.23
Psychiatric Diagnoses^b^ (Current)			
% Major Depressive Disorder	5.4%	11.4%	*x^2^* = 2.01, *p* = .16, *V* = 0.11
% Mania	2.2%	2.6%	*x^2^* = 0.02, *p* = .88, *V* = 0.01
% Anxiety Disorder^c^	13.0%	29.1%	***x^2^* = 6.74, *p* = .009, *V* = 0.20**
% Obsessive-Compulsive Disorder	2.2%	3.8%	*x^2^* = 0.40, *p* = .53, *V* = 0.05
% Eating Disorder	0%	1.3%	*x^2^* = 1.17, *p* = .28, *V* = 0.08
% Psychosis	1.1%	0%	*x^2^* = 0.86, *p* = .35, *V* = 0.07
Psychiatric Symptoms			
Childhood ADHD Symptoms (max = 9 ^d^)			
Inattention	4.00 (3.00)	3.25 (2.94)	*t* (169) = 1.64, *p* = .10, *d* = 0.25
Hyperactivity	4.32 (2.78)	3.61 (2.95)	*t* (169) = 1.61, *p* = .11, *d* = 0.25
Current ADHD Symptoms (max = 9 ^d^)			
Inattention	3.50 (3.04)	4.35 (3.38)	*t* (169) = −1.74, *p* = .08, *d* = 0.26
Hyperactivity	3.38 (2.83)	3.62 (2.78)	*t* (169) = −0.56, *p* = .58, *d* = 0.09
Mood/Anxiety (MASQ)			
General Distress Anxious Symptoms (max = 75)	17.73 (5.88)	20.42 (7.22)	***t* (169) = −2.69, *p* = .008, *d* = 0.41**
Anxious Arousal (max = 55)	23.77 (6.13)	25.87 (7.64)	***t* (169) = −2.00, *p* = .05, *d* = 0.30**
General Distress Depressive Symptoms (max = 85)	20.16 (7.86)	24.18 (9.57)	***t* (169) = −3.01, *p* = .003, *d* = 0.46**
Anhedonic Depression (max = 110)	55.72 (12.07)	59.72 (12.83)	***t* (169) = −2.10, *p* = .04, *d* = 0.32**
Personality (TIPI) (max = 14)			
Extraversion	9.37 (3.22)	9.57 (2.89)	*t* (169) = −0.43, *p* = .67, *d* = 0.07
Agreeableness	9.50 (2.30)	9.54 (2.42)	*t* (169) = −0.12, *p* = .90, *d* = 0.02
Conscientiousness	9.96 (2.37)	9.39 (3.34)	*t* (137.95) = 1.25, *p* = .21, *d* = 0.20
Emotional Stability	10.33 (2.49)	7.95 (3.00)	***t* (169) = 5.67, *p* <.001, *d* = 0.86**
Openness to Experience	11.28 (1.94)	11.67 (2.08)	*t* (169) = −1.26, *p* = .21, *d* = 0.19
Impulsive Behavior (UPPS-P)			
Urgency (max = 48)	28.12 (6.49)	28.52 (8.05)	*t* (149.42) = −0.35, *p* = .72, *d* = 0.05
Premeditation (max = 56)	22.01 (4.59)	23.47 (6.00)	*t* (144.83) = −1.76, *p* = .08, *d* = 0.31
Perseverance (max = 40)	20.32 (4.73)	21.94 (5.71)	***t* (169) = −2.03, *p* = .04, *d* = 0.31**
Sensation Seeking (max = 48)	38.82 (5.38)	34.29 (6.74)	***t* (169) = 4.88, *p* < .001, *d* = 0.74**
Positive Urgency (max = 56)	27.84 (8.92)	25.67 (9.14)	*t* (169) = 1.57, *p* = .12, *d* = 0.24

*Notes.* WTAR: Wechsler Test of Adult Reading; WASI: Wechsler Abbreviated Scale of Intelligence; MASQ: Mood and Anxiety Symptoms Questionnaire; TIPI: 10-Item Personality Inventory; UPPS-P: Impulsive Behavior Scale.

a WTAR/WASI.

b Includes SCID-5: Structured Clinical Interview for DSM-5; K-SADS: Schedule for Affective Disorders and Schizophrenia for School Aged Children; and MINI-Kid: MINI-International Neuropsychiatric Interview.

c Anxiety disorders: Gernalized Anxiety Disorder, Posttraumatic Stress Disorder, Panic Disorder, Agoraphobia, and Specific Phobia.

d A score of greater than or equal to 5 indicates symptoms consistent with ADHD.

**Table 2.** Substance use measures (means and standard deviations) by sex

	Males *n* = 92	Females *n* = 79	Statistic, *p*-value, Effect Size
Alcohol Use			
Age of Initiation (Years)	14.90 (1.89)	15.35 (1.85)	*t* (166) = −1.54, *p* = .13, *d* = 0.24
Dependence Symptoms (AUDIT)	7.82 (6.08)	6.04 (4.55)	***t* (166.04) = 2.18, *p* = .03, *d* = 0.33**
Alcohol Dependence (% Current)	27.2%	19.0%	*x^2^* = 1.59, *p* = .21, *V* = 0.10
Days Alcohol Consumed (past 90 days)	16.65 (15.06)	14.23 (13.87)	*U* = 3322.50, *p* = .34, *d* = 0.17
Drinks Consumed (past 90 days)	95.15 (112.78)	54.27 (66.05)	***U* = 2832.50, *p* = .01, *d* = 0.44**
Cannabis Use			
Age of Initiation (Years)	15.13 (1.97)	15.50 (2.03)	*t* (169) = −1.21, *p* = .23, *d* = 0.18
Age of Regular Use (Years)	16.63 (2.04)	17.13 (2.14)	*t* (155) = −1.41, *p* = .16, *d* = 0.24
Dependence Symptoms (CUDIT-R)	14.51 (5.27)	13.11 (5.30)	*t* (169) = 1.72, *p* = .09, *d* = 0.27
Cannabis Dependence (% Current)	46.7%	45.6%	*x^2^* = 0.02, *p* = 0.88, *V* = 0.01
CN-THCCOOH^a^	230.11 (340.65)	215.38 (457.46)	*U* = 2480.50, *p* = .07, *d* = 0.04
Days Cannabis Consumed (past 90 days)	59.37 (26.21)	47.90 (25.91)	*U* = 3084.50, *p* = .18, *d* = 0.44
Grams Consumed (past 90 days)	80.38 (103.99)	73.73 (135.52)	***U* = 2796.00, *p* = .009, *d* = 0.06**
Days Since Last Use	1.54 (1.65)	1.96 (2.66)	*t* (169) = −1.26, *p* = .21, *d* = 0.19
Lifetime (Cumulative) Cannabis Use			
<10 times	0.02%	0%	
10–50 times	0.05%	17.7%	
51–100 times	40.2%	43.0%	*x^2^* = 9.73, *p* = .05, *V* = 0.05
101–500 times	27.1%	24.1%	
501–100 times	25.0%	15.2%	
Withdrawal Symptoms			
Intensity (CWS)	29.71 (24.04)	36.24 (25.40)	*t* (168) = −1.72, *p* = .09, *d* = 0.26
Negative Impact (CWS)	26.89 (19.33)	34.28 (24.59)	***t* (145.10) = −2.15, *p* = .03, *d* = 0.33**
Nicotine Use			
Cigarettes Consumed (past 90 days)	69.00 (186.09)	6.92 (32.06)	***U* = 2412.00, *p* < .001, *d* = 0.46**
Days Since Last Use	18.05 (51.25)	24.81 (52.40)	*t* (129) = −0.73, *p* = .46, *d* = 0.19

*Notes*. AUDIT: Alcohol Use Disorders Identification Test; CUDIT-R: Cannabis Use Disorder Identification Test Revised; CWS: Cannabis Withdrawal Scale.

a Creatinine-adjusted 11-nor-9-carboxy-Δ9-tetrahydrocannabinol in ng/ml.

**Table 3.** Neuropsychological outcome measures (means and standard deviations) by sex

	Males *n* = 92	Females *n* = 79	Statistic, *p*-value, Effect Size
Memory			
Delayed Matching to Sample (DMS)^a^			
% Correct (Average of Delays)	70.40 (8.80)	66.71 (13.37)	*F* (1, 75) = 2.70, *p* = .11, *η_p_^2^* = 0.04
Correct Latency (simultaneous)	2829.92 (700.44)	2376.40 (627.03)	*F* (1, 75)=3.80, *p* = .06, *η_p_^2^* = 0.05
Correct Latency 4000 (delay) ms	3624.86 (976.37)	2938.86 (861.65)	***F* (1,75) = 6.80, *p* = .01, *η_p_^2^* = 0.08**
Correct Latency 12000 (delay) ms	4038.74 (1206.03)	3262.20 (1012.71)	***F* (1, 75) = 8.67, *p* = .004, *η_p_^2^* = 0.10**
Paired Associates Learning (PAL)^b^			
Total Errors (Adjusted)	5.40 (6.90)	6.90 (4.71)	*F* (1, 70) = 1.21, *p* = .28, *η_p_*^2^ = 0.02
First Trial Memory Score	16.29 (3.21)	15.02 (2.91)	*F* (1, 70) = 2.33, *p* = .13, *η_p_*^2^ = 0.03
Verbal Recognition Memory (VRM)^b^			
% Total Correct (Immediate)	32.69 (3.16)	33.17 (3.15)	*F* (1, 70) = 0.14, *p* = .71, *η_p_*^2^ = 0.02
% Total Correct (Delayed)	32.26 (2.80)	33.22 (2.06)	*F* (1, 70) = 1.43, *p* = .24, *η_p_*^2^ = 0.02
Attention/Executive Functions			
Multitasking Test (MTT)^c^			
Switching Cost ms	160.12 (113.11)	228.14 (127.50)	***F* (1, 147) = 12.23, *p* = .001, *η_p_*^2^ = 0.08**
Congruency Cost ms	43.22 (44.14)	41.70 (39.23)	*F* (1, 147) = 3.44, *p* = .07, *η_p_*^2^ = 0.02
Rapid Visual Information Processing (RVP)^c^			
A’	0.93 (0.05)	0.91 (0.05)	***F* (1, 157) = 6.52, *p* = .01, η_p_^2^ = 0.04**
Latency ms	443.75 (106.64)	474.79 (109.04)	*F* (1, 157) = 2.41, *p* = .12, *η_p_*^2^ = 0.02
One Touch Stockings of Cambridge (OTS)^c^			
Choices to Correct	1.35 (0.26)	1.44 (0.33)	*F* (1, 158) = 3.43, *p* = .07, *η_p_*^2^ = 0.02
Problems Solved	11.50 (2.01)	11.01 (2.18)	*F* (1, 158) = 3.11, *p* = .08, *η_p_*^2^ = 0.02
Spatial Working Memory (SWM)^b^			
Total Errors	4.69 (6.77)	7.14 (7.87)	*F* (1, 70) = 3.71, *p* = .06, *η_p_*^2^ = 0.05
Strategy	5.29 (2.63)	6.88 (2.45)	***F* (1, 70) = 7.67, *p* = .01, *η_p_*^2^ = 0.10**

*Notes*. Analyses are controlling for (as covariates): age, estimate of intelligence (IQ), amount (quantity) of alcohol and nicotine use in the past 90 days, mood and anxiety symptoms (MASQ subscales), emotional stability (TIPI subscale), and impulsive behavior (UPPS-P perseverance and sensation seeking subscales).

a Cohort 1, *n* = 88 (50 male, 38 female).

b Cohort 2, *n* = 83 (42 male, 41 female).

c Cohorts 1 and 2, *n* = 171 (79 male, 92 female).

**Table 4.** Main effects and interactions of age of cannabis initiation, amount of use, frequency of use, and withdrawal symptoms on neuropsychological outcome measures (β, *p*-values and 95% confidence intervals)

**Age of cannabis initiation**
Switching cost (MTT)
Sex: β = 1.43, *p* = .02 [61.77,649.39]
Age: β = 0.22, *p* = .02 [1.54,21.52]
Sex-by-age of initiation: β = −1.13, *p* = .07 [−36.60, 1.12]
Congruency cost (MTT)
IQ: β = 0.03, *p* = .04 [0.98,8.04]
Age: β = 0.25, *p* = .01 [0.98,8.04]
Immediate recall (VRM)
IQ: β = 0.30, *p* = .04 [0.003,0.14]
% Correct responses (DMS)
Perseverance: β = 0.31, *p* = .01 [1.42,11.30]
Correct latency simultaneous (DMS)
Anhedonic depression: β = 0.32, *p* = .02 [2.36,32.25]
Correct latency 4000ms delay (DMS)
Anhedonic depression: β = −0.48, *p* = .02 [−88.88,−9.58]
Depressive symptoms: β = 0.28, *p* = .04 [1.02,42.49]
Correct latency 12000ms delay (DMS)
Anhedonic depression: β = 0.30, *p* = .03 [2.50,52.82]
Depressive symptoms: β = −0.50, *p*= .01 [−110.66,−14.42]
Number of problems solved (OTS)
Perseverance: β = 0.19, *p* = .03 [0.01,0.14]
Anxious arousal: β = −0.25, *p* = .04 [−0.15,−0.01]
90 day cigarette use: β = −0.19, *p* = .03 [−0.01,0.00]
Mean choices to correct (OTS)
IQ: β = −0.26, *p* = .001 [β0.01,β0.003]
A’ (RVP)
90 day cigarette use: β = −0.19, *p* = .01 [0.00,0.000]
**Frequency of use**
Switching cost (MTT)
IQ: β = −0.22, *p* = .01 [−1.42,−0.18]
Total errors (SWM)
IQ: β = −0.27, *p* = .04 [−0.29,−0.01]
Search strategy (SWM)
Sex: β = 0.60, *p* = .03 [0.33,5.97]
Immediate recall (VRM)
IQ: β = 0.28, *p* = .03 [0.01,0.13]
% Correct responses (DMS)
IQ: β = 0.25, *p* = .03 [0.33,5.74]
Age: β = 0.26, *p* = .03 [1.40,26.16]
Perseverance: β = 0.35, *p* = .01 [1.95,12.54]
Correct latency simultaneous (DMS)
Anhedonic depression: β = 0.33, *p* = .02 [2.99,33.26]
Correct latency 4000ms delay (DMS)
Anhedonic depression, β = 0.31, *p* = .03 [2.70,44.24]
Depressive symptoms, β = −0.47, *p* = .02 [−88.19,−9.76]
Correct latency 12000ms delay (DMS)
Anhedonic depression, β = 0.32, *p* = .02 [4.30,55.05]
Depressive symptoms, β = −0.50, *p* = .01 [−109.59,−13.76]
Number of problems solved (OTS)
Perseverance: β = 0.18, *p* = .04 [0.04,1.39]
90 day cigarette use: β = −0.19, *p* = .02 [−0.01,0.00]
Mean choices to correct (OTS)
IQ: β = −0.28, *p* < .001[−0.01,−0.003]
Anxious arousal: β = 0.24, *p* = .04 [0.00,0.02]
**Negative impact of withdrawal**
Mean choices to correct (OTS)
IQ: β = −0.33, *p* < .001 [−0.01,−0.01]
Anxious arousal: β = 0.32, *p* = .01 [0.004,0.02]
Sex-by-negative impact: β = 0.40, *p* = .02 [0.001, 0.009]
Number of problems solved (OTS)
IQ: β = 0.19, *p* = .03 [0.004,0.07]
90 day cigarette use: β = −0.20, *p* = .01 [−0.01,−0.001]
Anxious arousal: β = −0.32, *p* = .01 [−0.17,−0.02]
Perseverance: β = 0.22, *p* = .02 [0.02,0.16]
Sex-by-negative impact: β = −0.40, *p* = .02 [−0.06, −0.01]
Congruency cost (MTT)
IQ: β = −0.21, *p* = .02 [−1.45, −0.13]
Total errors (SWM)
Sex: β = 0.38, *p* = .03 [0.62,10.65]
Strategy (SWM)
Sex: β = 0.42, *p* = .02 [0.34,4.07]
Immediate recall (VRM)
IQ: β = 0.41, *p* = .01 [0.03,0.17]
Delayed recall (VRM)
IQ: β = 0.36, *p* = .02 [0.01,0.13]
% Correct responses (DMS)
IQ: β = 0.33, *p* = .01 [1.23,6.69]
Perseverance: β = 0.28, *p* = .02 [0.91,10.69]
Negative impact: β = 0.52, *p* = .004 [0.99,4.84]
Correct latency simultaneous (DMS)
Anhedonic depression: β = 0.32, *p* = .02 [2.49,32.65]
Correct latency 4000ms delay (DMS)
Anhedonic depression: β = 0.28, *p* = .04 [1.22,42.23]
Depressive symptoms: β = −0.54, *p* = .01 [−95.35,−15.42]
Correct latency 12000ms delay (DMS)
Anhedonic depression: β = 0.30, *p* = .03 [2.70,53.43]
Depressive symptoms: β = −0.49, *p* = .02 [−110.87,−12.00]
Response latency (RVP)
IQ: β = −0.19, *p* = .02 [−3.39,−0.28]
Age: β = −0.22, *p* = .01 [−18.19,−2.17]
Negative impact: β = −0.46, *p* = .001 [−3.53,−0.96]
A’ (RVP)
IQ: β = 0.37, *p* < .001 [0.001,0.002]
Age: β = 0.17, *p* = .04 [0.00,0.01]
90 day cigarette use: β = −0.19, *p* = .01 [0.00,0.00]
Anxious arousal: β = −0.25, *p* = .03 [−0.003,0.00]
Emotional stability: β = 0.18, *p* = .04 [0.00,0.01]
**Intensity of withdrawal**
Switching cost (MTT)
Sex: β = 0.32, *p* = .03 [9.82,149.06]
Congruency cost (MTT)
IQ: β = −0.23, *p* = .01 [−1.52, −0.21
Total errors (SWM)
Sex: β = 0.42, *p* = .04 [0.21,12.02]
Strategy (SWM)
Sex: β = 0.52, *p* = .02 [0.53,4.94]
Immediate recall (VRM)
IQ: β = 0.45, *p* = .003 [0.04,0.18]
Intensity: β = 0.49, *p* = .03 [0.01,0.11]
Response latency (RVP)
Age: β = −0.29, *p* = .002 [−21.01,−4.91]
A’ (RVP)
IQ: β = 0.33, *p* < .01 [0.001,0.002]
Age: β = 0.21, *p* = .01[0.01,0.01]
90 day cigarette use: β = −0.20, *p* = .01[0.00,0.00]
**Amount of use**
Response latency (RVP)
Age: β = −0.27, *p* < .001 [597.06,1143.49]
Sex-by-amount of use: β = 0.28, *p* = .02 [0.05, 0.55]
First trial memory score (PAL)
Sex: β = −0.48, *p* = .002 [−4.84,−1.10]
Amount of use: β = −0.64, *p* = .001 [−0.02,−0.01]
Sex-by-amount of use: *p* = 0.68, *p* = .001 [0.01,0.03]
Total errors (PAL)
Sex: β = 0.46, *p* = .002 [2.18,8.84]
Amount of use: β = 0.88, *p* < .001 [0.02, 0.05]
Sex-by-amount of use: β = −0.78, *p* < .001 [−0.05,−0.02]
Delayed recall (VRM)
IQ: β = 0.37, *p* = .02 [0.01,0.13]
% Correct responses (DMS)
Age: β = 0.25, *p* = .02 [1.87,24.75]
IQ: β = 0.29, *p* = .01 [0.74,6.06]
Perseverance: β = 0.31, *p* = .01 [1.47,11.39]
Intensity: β = 0.42, *p* = .01 [0.47,3.54]
Correct latency simultaneous (DMS)
Anhedonic depression: β = 0.29, *p* = .04
Correct latency 4000ms delay (DMS)
Depressive symptoms: β = −0.57, *p* = .004 [−97.92,−18.84]
Correct latency 12000ms delay (DMS)
Anhedonic depression: β = 0.28, *p* = .04 [0.80,51.74]
Depressive symptoms: β = −0.52, *p* = .01 [−113.77,−15.19]
Number of problems solved (OTS)
IQ: β = 0.19, *p* = .02 [0.01,0.07]
90 day cigarette use: β = −0.19, *p* = .02 [−0.001,0.00]
Anxious arousal: β = −0.30, *p* = .02 [−0.17,−0.02]
Perseverance: β = 0.19, *p* = .03 [0.01,0.15]
Mean choices to correct (OTS)
IQ: β = −0.33, *p* < .001 [−0.1,−0.01]
Anxious arousal: β = 0.30, *p* = 0.01 [0.003,0.02]
Mean latency (RVP)
Age: β = −0.29, *p* = .002 [−21.08,−5.05]
A’ (RVP)
Age: β = 0.20, *p* = .02 [0.001,0.01]
IQ: β = 0.35, *p* < .001 [0.001,0.002]
90 day cigarette use: β = −0.20, *p* = .01 [0.00,0.00]
Anxious arousal: β = −0.26, *p* = .02 [−0.004,0.00]
Emotional stability: β = 0.18, *p* = .04 [0.00,0.01]

*Notes:* DMS: Delayed Matching to Sample; PAL: Paired Associates Learning; VRM: Verbal Recognition Memory; MTT: Multitasking Test; RVP: Rapid Visual Information Processing; OTS: One Touch Stockings of Cambridge; SWM: Spatial Working Memory

## Footnotes

1 Similar to objective methods employed by other research teams (e.g. Hindocha, Freeman, & Curran, 2017), participants were asked to estimate the typical amount of cannabis used per use occasion. A cannabis placebo sample (i.e. oregano) was weighed to the nearest hundredth gram with a Pocket-Size scale. This estimate was then multiplied by the number of use occasions to estimate the amount of cannabis exposure over the past 90 days.
